# Statement of Retraction: Circ_0005320 promotes oral squamous cell carcinoma tumorigenesis by sponging microRNA-486-3p and microRNA-637

**DOI:** 10.1080/21655979.2024.2299555

**Published:** 2024-02-20

We, the Editors and Publisher of the journal *Bioengineered*, have retracted the following article:

Xiaotao Zheng, Fang Du, Xuepeng Gong & Ping Xu (2022) Circ_0005320 promotes oral squamous cell carcinoma tumorigenesis by sponging microRNA-486-3p and microRNA-637, Bioengineered, 13:1, 440-454, DOI: 10.1080/21655979.2021.2009317

Since publication, significant concerns have been raised about the integrity of the data and reported results in the article. When approached for an explanation, the authors requested the article be retracted due to consent issues. As verifying the validity of published work is core to the integrity of the scholarly record, we are therefore retracting the article. All authors listed in this publication have been informed. The authors have agreed to retract the article.

We have been informed in our decision-making by our policy on publishing ethics and integrity and the COPE guidelines on retractions.

The retracted article will remain online to maintain the scholarly record, but it will be digitally watermarked on each page as ‘Retracted’.

